# Crystal structure and stable property of the cancer-associated heterotypic nucleosome containing CENP-A and H3.3

**DOI:** 10.1038/srep07115

**Published:** 2014-11-19

**Authors:** Yasuhiro Arimura, Kazuyoshi Shirayama, Naoki Horikoshi, Risa Fujita, Hiroyuki Taguchi, Wataru Kagawa, Tatsuo Fukagawa, Geneviève Almouzni, Hitoshi Kurumizaka

**Affiliations:** 1 Laboratory of Structural Biology, Graduate School of Advanced Science and Engineering, Waseda University, 2-2 Wakamatsu-cho, Shinjuku-ku, Tokyo 162-8480, Japan; 2 Program in Chemistry and Life Science, School of Science and Engineering, Meisei University, 2-1-1 Hodokubo, Hino-shi, Tokyo 191-8506, Japan; 3 Department of Molecular Genetics, National Institute of Genetics, Mishima, Shizuoka 411-8540, Japan; 4 Institut Curie, Centre de Recherche, Paris, F-75248 France; 5 CNRS, UMR3664, Paris, F-75248 France

## Abstract

The centromere-specific histone H3 variant, CENP-A, is overexpressed in particular aggressive cancer cells, where it can be mislocalized ectopically in the form of heterotypic nucleosomes containing H3.3. In the present study, we report the crystal structure of the heterotypic CENP-A/H3.3 particle and reveal its “hybrid structure”, in which the physical characteristics of CENP-A and H3.3 are conserved independently within the same particle. The CENP-A/H3.3 nucleosome forms an unexpectedly stable structure as compared to the CENP-A nucleosome, and allows the binding of the essential centromeric protein, CENP-C, which is ectopically mislocalized in the chromosomes of CENP-A overexpressing cells.

In eukaryotes, chromatin compacts genomic DNA with the nucleosome, as the basic repeating unit. Histones H2A, H2B, H3, and H4 are the protein components of the nucleosome. The histone octamer, containing two each of histones H2A, H2B, H3, and H4, left-handedly wraps about 150 base pairs of DNA around itself in the nucleosome^1^,^2^. In addition to the canonical types of histones, non-allelic isoforms of histones H2A, H2B, and H3 have been identified as histone variants in many species^3^, and are suggested to have specific functions in the regulation and maintenance of genomic DNA in chromatin^4^,^5^,^6^.

In humans, eight types of histone H3 genes, encoding H3.1, H3.2, H3.3, H3T (also named H3.4), H3.5, H3.X, H3.Y, and CENP-A (also named CenH3), have been identified^3^,^7^. H3.1, H3.2, and H3.3 are constantly produced in somatic cells. H3.1 and H3.2 are the canonical types of histone H3, and are expressed in the S-phase of the cell cycle^8^. On the other hand, H3.3 is continuously produced throughout the cell cycle. During the transcription and DNA repair processes, H3.3 functions as a replacement histone that is incorporated into chromatin regions by the HIRA histone chaperone, after the depletion of the canonical H3.1^8^,^9^,^10^. In addition, H3.3 is specifically localized in the telomeric and pericentromeric regions of chromosomes, by a complex containing the histone chaperone DAXX and the nucleosome remodeler ATRX proteins^11^,^12^. Therefore, H3.3 may have dual functions as a replacement histone and an architectural component of distinct chromosome domains.

Among the histone H3 variants, CENP-A is the most distant isoform, and is conserved from yeasts to humans. CENP-A specifically localizes to centromeres and epigenetically specifies the sites for kinetochore assembly^13^,^14^,^15^,^16^,^17^,^18^. CENP-A expression is tightly controlled in normal cells, and its chromosome localization is strongly restricted within centromeric regions. However, CENP-A is reportedly overexpressed in particular aggressive cancer cells^4^,^19^,^20^,^21^,^22^. In *Drosophila* cells, overexpressed CENP-A (CID) is mislocalized into noncentromeric euchromatic regions and leads mitotic delays, anaphase bridges and chromosome fragmentation^23^. Similarly, in human cells, CENP-A overexpression can lead to its ectopic localization to chromosome regions with active histone turnover, as shown in cancer cell lines^19^,^24^. At these ectopic loci, CENP-A forms heterotypic nucleosomes, containing one each of the histone H3 variants, CENP-A and histone H3.3, within a single nucleosome^24^. These heterotypic nucleosomes occlude CTCF binding, and their presence may increase DNA damage tolerance in cancer cells^24^. However, the structural features of the heterotypic CENP-A/H3.3 nucleosome are unknown.

In the present study, we report the crystal structure of the human CENP-A/H3.3 nucleosome at 2.67 Å resolution. The CENP-A/H3.3 nucleosome forms a “hybrid structure”, in which the physical characteristics of CENP-A and H3.3 are independently conserved within the same nucleosome. Unexpectedly, we found that the heterotypic CENP-A/H3.3 nucleosome is more stable than the CENP-A nucleosome, and its stability is very similar to that of the H3.3 nucleosome. In addition, the CENP-A/H3.3 nucleosome retains the ability to bind to the essential centromeric protein, CENP-C, whose ectopic localization may also be harmful for the proper regulation of cell division and chromosome integrity.

## Results and Discussion

### Preparation of the heterotypic CENP-A/H3.3 nucleosome

**Figure 1:**
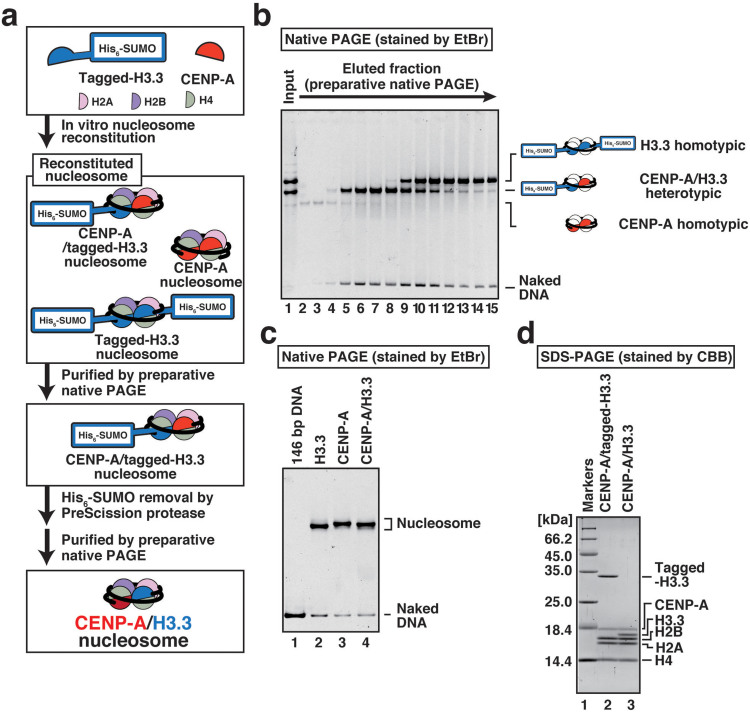
Preparation of the CENP-A/H3.3 nucleosome. (a) Schematic representation of the CENP-A/His_6_-SUMO-H3.3 nucleosome preparation and the CENP-A/H3.3 nucleosome preparation with the His_6_-SUMO removal step. The CENP-A and His_6_-SUMO-H3.3 molecules are colored red and blue, respectively. (b) The reconstituted nucleosomes (lane 1) were separated by native PAGE with the Prep Cell apparatus. Purified nucleosome fractions were analyzed by 6% native PAGE with ethidium bromide staining. The fractions shown in lanes 5–8 were collected. (c) The purified H3.3, CENP-A, and CENP-A/H3.3 nucleosomes were analyzed by 6% PAGE with ethidium bromide staining. (d) The protein compositions of the CENP-A/His_6_-SUMO-H3.3 nucleosome (lane 2) and the CENP-A/H3.3 nucleosome (lane 3) were analyzed by 18% SDS-PAGE with Coomassie Brilliant Blue staining.

To decipher its structural features, we reconstituted the heterotypic CENP-A/H3.3 nucleosome using purified histones. For this purpose, we established a method to prepare this heterotypic nucleosome (Fig. 1a), based on the method for the heterotypic nucleosome reconstitution with yeast H2A and FLAG-tagged HtZ^25^. Human histones H2A, H2B, H3.3, H4, and CENP-A were prepared as described previously^26^,^27^, and histone H3.3 was also prepared as a His_6_-SUMO tagged protein expressed in bacteria (Supplementary Fig. 1a). The nucleosomes were then reconstituted on a 146 base-pair palindromic DNA^1^ by the salt-dialysis method (Fig. 1b, lane 1). In this procedure, three types of nucleosomes were simultaneously formed: two homotypic nucleosomes with either two CENP-As or two His_6_-SUMO-H3.3s, and a heterotypic nucleosome containing one CENP-A and one His_6_-SUMO-H3.3. In non denaturing polyacrylamide gel electrophoresis (native PAGE), the heterotypic CENP-A/His_6_-SUMO-H3.3 nucleosome migrated faster than the homotypic His_6_-SUMO-H3.3/His_6_-SUMO-H3.3 nucleosome, but slower than the homotypic CENP-A/CENP-A nucleosome, which allowed us to isolate the CENP-A/His_6_-SUMO-H3.3 nucleosome by preparative native PAGE (Fig. 1b). After the purification, the His_6_-SUMO portion of the His_6_-SUMO-H3.3 fusion protein was proteolytically removed by PreScission protease treatment (Supplementary Fig. 1b, lanes 1–2). We further purified the heterotypic CENP-A/H3.3 nucleosome by a second round of preparative native PAGE (Fig. 1c and Supplementary Fig. 1b). The purified heterotypic CENP-A/H3.3 nucleosome contained all five histones, H2A, H2B, H3.3, H4, and CENP-A (Fig. 1d).

### The structural characteristics of CENP-A and H3.3 are conserved independently within the heterotypic CENP-A/H3.3 nucleosome

**Figure 2:**
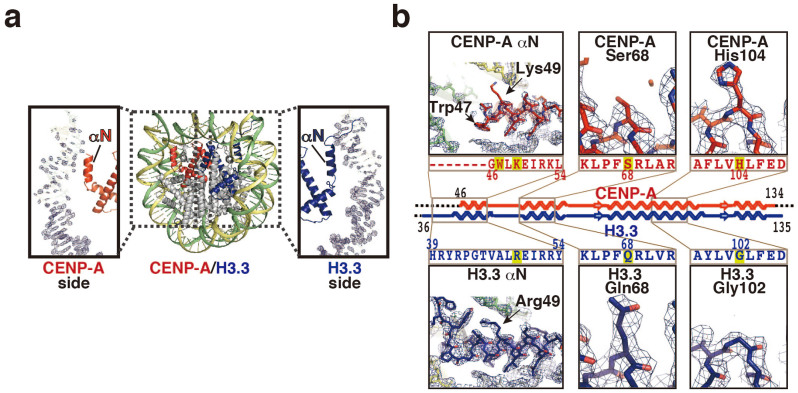
Crystal structure of the human CENP-A/H3.3 nucleosome. (a) The CENP-A/H3.3 nucleosome structure is presented. CENP-A and H3.3 molecules are colored red and blue, respectively. The *2mFo - DFc* maps of the two DNA end regions of the CENP-A/H3.3 were calculated and contoured at the 2.0σ level. (b) Close-up views of the CENP-A αN helix, the H3.3 αN helix, the CENP-A Ser68 residue, the H3.3 Gln68 residue, the CENP-A His104 residue, and the H3.3 Gly102 residue. Electron density maps are presented at the 1.5σ level.

We then determined the crystal structure of the heterotypic CENP-A/H3.3 nucleosome at 2.67 Å resolution (Fig. 2a and Supplementary Table 1). In the crystal structure, the CENP-A and H3.3 molecules were clearly distinguishable. For example, the side chain moiety of the CENP-A-specific His104 residue, corresponding to the H3.3 Gly102 residue, was clearly observed in the CENP-A molecule in the heterotypic nucleosome (Fig. 2b and Supplementary Fig. 2b and c). On the other hand, the H3.3-specific Gln68 residue, corresponding to the CENP-A Ser68 residue, was distinctly visible in the H3.3 molecule (Fig. 2b and Supplementary Fig. 2b and c). In the homotypic CENP-A nucleosome, the CENP-A αN helices were shorter than the canonical H3.1 αN helices in H3.1 homotypic nucleosomes^27^. This CENP-A-specific αN structure is perfectly conserved in the CENP-A/H3.3 nucleosome, in which the CENP-A αN helix is one helical turn shorter than the H3.3 αN helix (Fig. 2b and Supplementary Fig. 2a).

**Figure 3:**
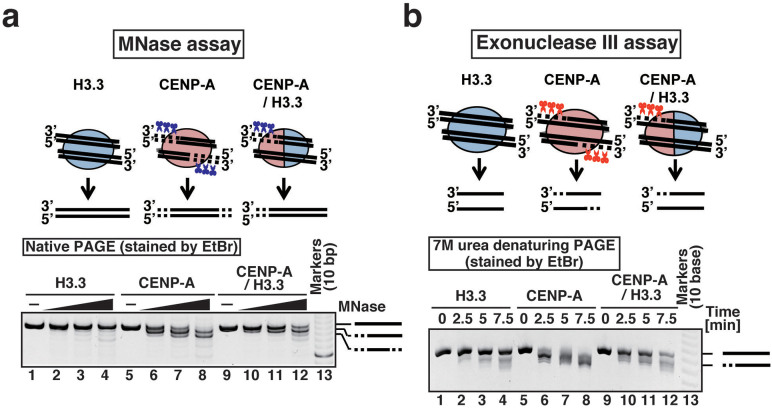
The DNA end close to CENP-A is asymmetrically flexible in the CENP-A/H3.3 nucleosome. (a) MNase assay. The H3.3, CENP-A, and CENP-A/H3.3 nucleosomes were treated with MNase (0, 0.3, 0.5 and 0.7 units), and the resulting DNA fragments were analyzed by native PAGE. The gel image shown is a representative of four independent experiments, in which similar results were obtained. (b) ExoIII assay. The H3.3, CENP-A, or CENP-A/H3.3 nucleosomes were incubated with or without 2.0 units of ExoIII for 2.5, 5 and 7.5 minutes at 37°C, and the resultant DNA fragments were extracted and analyzed by 14% denaturing PAGE with 7 M urea. The gel image shown is a representative of three independent experiments, in which similar results were obtained.

The structural asymmetry of the CENP-A and H3.3 molecules induces the asymmetric wrapping of the DNA in the CENP-A/H3.3 nucleosome (Fig. 2a). The different electron densities observed at the two ends suggested that the DNA end on the CENP-A side is more flexible than that on the H3.3 side in the CENP-A/H3.3 nucleosome. This was confirmed by differential micrococcal nuclease (MNase) digestion at the ends (Fig. 3a and Supplementary Fig. 3a) and by a treatment with Exonuclease III (ExoIII), which digests one strand of duplex DNA from the 3′ end, and degraded only one end of the DNA in the CENP-A/H3.3 nucleosome (Fig. 3b and Supplementary Fig. 3b). In contrast, with the homotypic CENP-A nucleosome (Fig. 3a and Supplementary Fig. 3a), MNase attacked about 20 base pairs of DNA (Fig. 3a and Supplementary Fig. 3a), and ExoIII degraded about 10 bases from both DNA ends (Fig. 3b and Supplementary Fig. 3b).

Therefore, in the CENP-A/H3.3 nucleosome, the DNA end close to CENP-A could be more flexible than the DNA end close to H3.3. These results are consistent with a previous *in vivo* MNase analysis, which demonstrated that the average DNA length tightly wrapped within the CENP-A/H3.3 nucleosomes is shorter than that of the canonical H3 nucleosome, but longer than that of the homotypic CENP-A nucleosome^24^.

### The heterotypic CENP-A/H3.3 nucleosome is more stable than the homotypic CENP-A nucleosome

**Figure 4:**
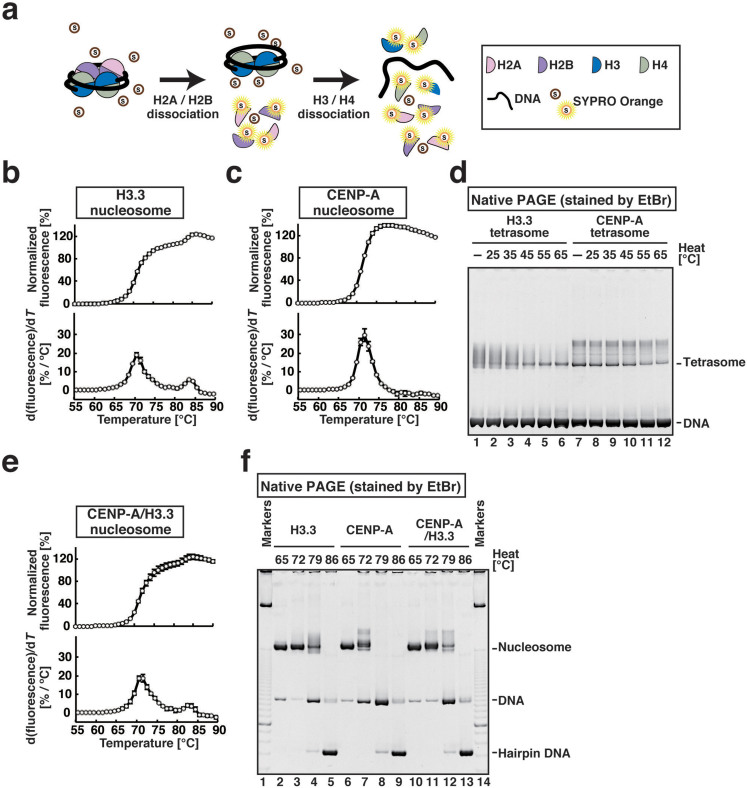
Stability of the CENP-A/H3.3 nucleosome. (a) Schematic representation of the thermal stability assay. (b) Thermal stability curve of the H3.3 nucleosome. The fluorescence intensity was plotted against each temperature (from 55°C to 90°C). The derivative values of the H3.3 stability curve presented in the upper panel are plotted against the temperatures (bottom panel). Means ± s.d. (*n* = 3) are shown. (c) A thermal stability curve of the CENP-A nucleosome. The fluorescence intensity was plotted against each temperature (from 55°C to 90°C). The derivative values of the CENP-A stability curve presented in the upper panel are plotted against the temperatures (bottom panel). Means ± s.d. (*n* = 3) are shown. (d) Tetrasomes, reconstituted with the H3.3-H4 tetramer or the CENP-A-H4 tetramer and DNA, were analyzed by 6% native PAGE. Lane 1 indicates the H3.3 tetrasome before incubation. Lanes 2, 3, 4, 5, and 6 indicate the H3.3 tetrasomes after 25°C, 35°C, 45°C, 55°C, and 65°C incubations, respectively. Lane 7 indicates the CENP-A tetrasome before incubation. Lanes 8, 9, 10, 11, and 12 indicate the CENP-A tetrasomes after 25°C, 35°C, 45°C, 55°C, and 65°C incubations, respectively. DNA was visualized by ethidium bromide staining. The gel image is a representative of seven independent experiments with similar results. (e) A thermal stability curve of the CENP-A/H3.3 nucleosome. The fluorescence intensity was plotted against each temperature (from 55°C to 90°C). The derivative values of the CENP-A/H3.3 stability curve presented in the upper panel are plotted against the temperatures (bottom panel). Means ± s.d. (*n* = 3) are shown. (f) The H3.3, CENP-A, and CENP-A/H3.3 nucleosomes were incubated for 1 min at each temperature from 25°C, and the samples at 65°C, 72°C, 79°C, and 86°C were analyzed by 6% native PAGE. DNA was visualized by ethidium bromide staining. The gel image is a representative of three independent experiments with similar results.

To assess the biological significance of the CENP-A/H3.3 nucleosome, we examined its thermal stability. For this, we utilized a fluorescent dye, SYPRO Orange, which binds to denatured proteins by hydrophobic interactions^28^. In the assay, the fluorescence signal from SYPRO Orange was monitored as a function of increasing temperature. An increase in the fluorescence signal indicates that the histones have dislodged from the nucleosome and become denatured^29^,^30^ (Fig. 4a).

For the H3.3 nucleosome, a biphasic histone dissociation curve (Fig. 4b, upper panel) was observed, with first and second Tm values of 70–71°C and 83–84°C, respectively (Fig. 4b, lower panel). The first and second thermal dissociation curves reflect the stepwise dissociation of the H2A-H2B dimer and the H3.3-H4 tetramer, respectively^30^. The CENP-A nucleosome exhibited a monophasic dissociation curve (Fig. 4c, upper panel) with a Tm value of about 71–72°C (Fig. 4c, lower panel). This suggested that the CENP-A-H4 tetramer may be unstably incorporated into the nucleosome, as compared to the H3.3-H4 tetramer, and may simultaneously dissociate with the H2A-H2B dimer.

To test the unstable association of the CENP-A-H4 tetramer with DNA, we reconstituted tetrasomes, containing the CENP-A-H4 tetramer or the H3.3-H4 tetramer with a 146 base-pair DNA fragment (CENP-A tetrasome and H3.3 tetrasome, respectively), in the absence of the H2A-H2B dimer. After the salt dialysis reconstitution, the tetrasomes were fractionated by native PAGE. The bands corresponding to the H3.3 tetrasomes were smeared, probably by the unusual histone-DNA binding and/or the multiple positions of the H3.3-H4 tetramer in the tetrasomes (Fig. 4d, lanes 1–3). The H3.3 tetrasomes ran as a single band, when the sample was treated at 45–65°C for 2 hr (Fig. 4d, lanes 4–6), suggesting the formation of the stably positioned H3.3 tetrasome. In contrast, the CENP-A tetrasomes had a tendency to form positioned tetrasomes at lower temperatures (Fig. 4d, lanes 7–10), but the positioned CENP-A tetrasomes were substantially disrupted by the 55–65°C treatment (Fig. 4d, lanes 11–12). These results confirmed that the association of the CENP-A-H4 tetramer with DNA was less stable than that of the H3.3-H4 tetramer. Therefore, the homotypic CENP-A nucleosome ectopically mislocalized in the chromosome arms could easily be removed in cells, although it should be stably maintained with additional proteins in functional centromeres.

Surprisingly, although the DNA end is asymmetrically detached in the CENP-A/H3.3 nucleosome, we found that the CENP-A/H3.3 nucleosome was very stable, in contrast to the CENP-A nucleosome (Fig. 4e). The CENP-A/H3.3 nucleosome generated a biphasic histone dissociation curve (Fig. 4e, upper panel), and the first and second Tm values (70–72°C and 82–84°C) were very similar to those of the H3.3 nucleosome (Fig. 4e, lower panel). Consistently, a gel retardation assay revealed that the CENP-A nucleosome was disrupted at lower temperatures (72 and 79°C), as compared to the H3.3 and CENP-A/H3.3 nucleosomes (Fig. 4f).

### The CENP-C fragment binds to the heterotypic CENP-A/H3.3 nucleosome

**Figure 5:**
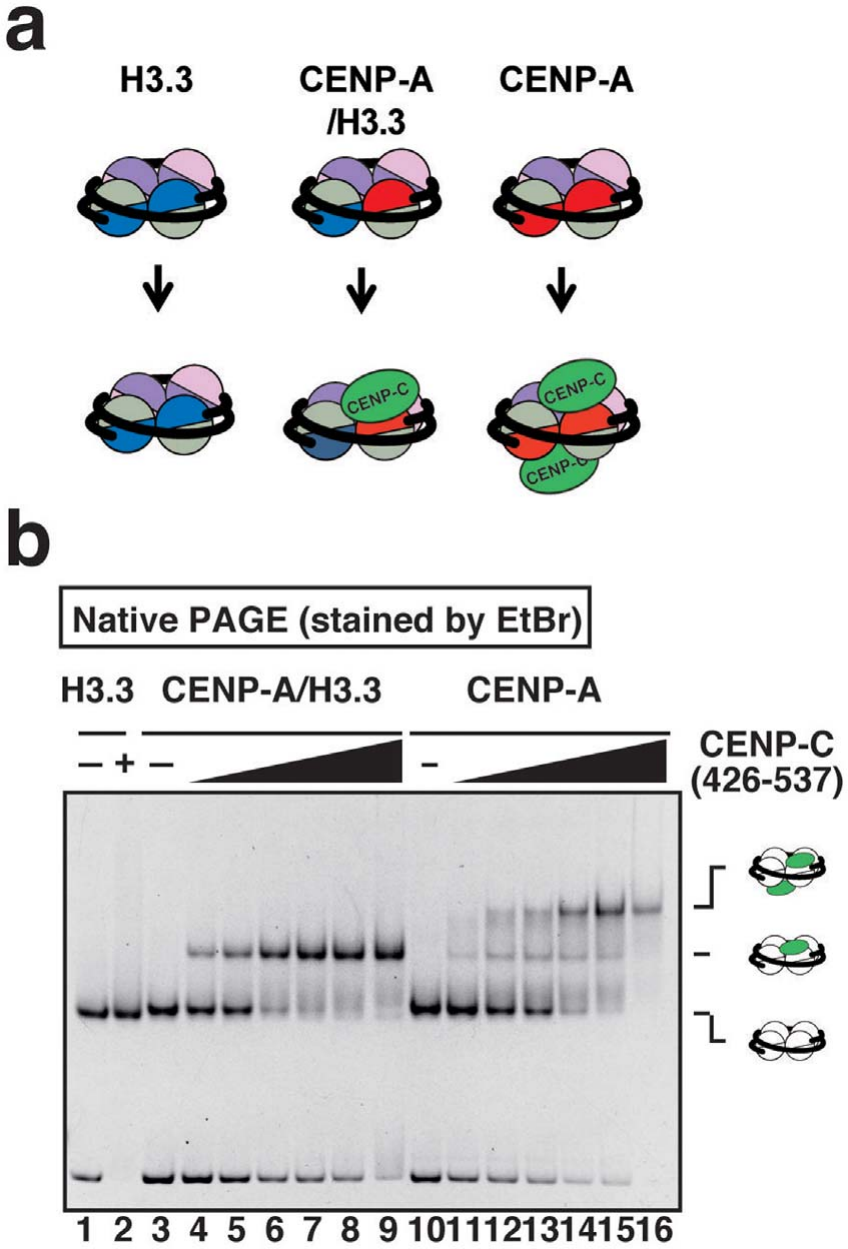
CENP-C binding to the CENP-A/H3.3 nucleosome. (a) Schematic representations of CENP-C binding to the H3.3 nucleosome, the CENP-A nucleosome, and the CENP-A/H3.3 nucleosome. (b) The binding of CENP-C to the CENP-A/H3.3 nucleosome. CENP-C(426–537) peptide binding to the H3.3 nucleosome (0.2 μM), the CENP-A nucleosome (0.2 μM), and the CENP-A/H3.3 nucleosome (0.2 μM) was evaluated by a gel mobility shift assay. The CENP-C(426–537) peptide concentrations are 0 μM (lanes 1, 3, and 10), 0.2 μM (lanes 4 and 11), 0.4 μM (lanes 5 and 12), 0.6 μM (lanes 6 and 13), 0.8 μM (lanes 2, 7, and 14), 1.0 μM (lanes 8 and 15), and 1.2 μM (lanes 9 and 16). The gel image is a representative of three independent experiments with similar results.

We next tested the binding of CENP-C to the CENP-A/H3.3 nucleosome. The CENP-C(426–537) fragment (a CENP-A binding region)^31^,^32^,^33^,^34^ efficiently bound to the CENP-A/H3.3 nucleosome (Fig. 5a and b). As anticipated, only one CENP-C bound to the CENP-A/H3.3 nucleosome (Fig. 5b, lanes 3–9), while two CENP-C peptides bound to the homotypic CENP-A nucleosome (Fig. 5b, lanes 10–16), and no CENP-C binding was detected with the homotypic H3.3 nucleosome (Fig. 5b, lanes 1 and 2).

## Conclusion

We conclude that the structural characteristics of CENP-A and H3.3 are conserved in the CENP-A/H3.3 nucleosome, which forms a surprisingly stable structure. This stable existence of the CENP-A/H3.3 nucleosome may cause ectopic kinetochore assembly, which could lead to neocentromere formation and chromosome instability in cancer cells^19^,^20^,^21^,^22^,^23^,^24^. The unique structural and physical properties of the CENP-A/H3.3 nucleosome provide important insights toward understanding the chromosome instability observed in cancer progression, thus offering a basis for potential drug development.

## Methods

### Purification of recombinant human histones

The DNA fragment encoding human histone H3.3 was inserted between the *NdeI* and *BamHI* sites of the pET21a-NHSP2 vector^35^, in which the His_6_-tag sequence, the *Saccharomyces cerevisiae* SUMO homolog, Smt3, and a PreScission protease cleavage sequence are located just upstream of the H3.3 coding sequence. Then, the N-terminally His_6_-SUMO tagged H3.3 was expressed in *Escherichia coli* BL21(DE3) cells in the presence of isopropyl-β-D-thiogalactopyranoside (final concentration 1 mM). His_6_-SUMO tagged H3.3 was recovered from inclusion bodies with 50 mM Tris-HCl (pH 8.0) buffer, containing 7 M guanidine hydrochloride, 500 mM NaCl, 1 mM phenylmethylsulfonyl fluoride, and 5% glycerol, and was purified by nickel-nitrilotriacetic acid (Ni-NTA) agarose chromatography (Qiagen) under denaturing conditions with 6 M urea. His_6_-SUMO tagged H3.3 was then purified by Mono S column chromatography (GE Healthcare) under denaturing conditions with 6 M urea. The purified His_6_-SUMO tagged H3.3 was dialyzed against water containing 2 mM 2-mercaptoethanol, freeze-dried, and stored at 4°C.

Human histones H2A, H2B, H3.3, CENP-A, and H4 were expressed and purified as described previously^26^,^27^,^36^.

### Preparation of the histone octamer containing H2A, H2B, His_6_-SUMO-H3.3, CENP-A, and H4

Purified histones H2A, H2B, His_6_-SUMO-H3.3, CENP-A, and H4 were mixed in a 1:1:0.6:0.4:1 ratio, and the histone octamer was reconstituted in 20 mM Tris-HCl (pH 7.5) buffer, containing 7 M guanidine-HCl and 20 mM 2-mercaptoethanol. To curtail the formation of the histone octamer containing two CENP-A molecules, we reduced the amount of CENP-A relative to His_6_-SUMO-H3.3 in the histone octamer reconstitution mixture. The guanidine-HCl was removed by dialysis against 10 mM Tris-HCl (pH 7.5) buffer, containing 1 mM EDTA, 5 mM 2-mercaptoethanol, and 2 M NaCl (500 ml) for 4 hr, and this dialysis step was repeated four times. The resulting mixtures of histone octamers containing two CENP-A, two His_6_-SUMO-H3.3, or one each of CENP-A and His_6_-SUMO-H3.3 were further purified by Superdex 200 gel filtration chromatography.

### Preparation of the heterotypic nucleosome containing CENP-A and H3.3

The nucleosome reconstitution was performed with the histone octamers prepared above in the presence of the 146 base-pair palindromic α-satellite DNA derivative^1^, by the salt dialysis method. Three nucleosomes were thus formed: 1) the homotypic His_6_-SUMO-H3.3 nucleosome, 2) the homotypic CENP-A nucleosome, and 3) the heterotypic CENP-A/His_6_-SUMO-H3.3 nucleosome. These three nucleosomes were separated by native PAGE, and the heterotypic CENP-A/His_6_-SUMO-H3.3 nucleosome was purified by preparative native PAGE (Fig. 1b). The His_6_-SUMO portion of H3.3 was then proteolytically removed by PreScission protease (GE Healthcare) (Supplementary Fig. 1b, lanes 1–2), and the heterotypic CENP-A/H3.3 nucleosome was further purified by preparative native PAGE (Supplementary Fig. 1b, lanes 3–12). The purified heterotypic CENP-A/H3.3 nucleosome and its histone composition were analyzed by native PAGE (Fig. 1c, lane 4) and SDS-PAGE (Fig. 1d, lane 3).

### Crystallization and structure determination

The purified CENP-A/H3.3 nucleosome was dialyzed against 20 mM potassium cacodylate (pH 6.0) buffer, containing 1 mM EDTA, and 1 μl of the 3 mg/ml CENP-A/H3.3 nucleosome solution (concentration of DNA) was mixed with 1 μl of 20 mM potassium cacodylate (pH 6.0) buffer, containing 50 mM KCl and 90 mM MnCl_2_. The sample was equilibrated against a reservoir solution of 20 mM potassium cacodylate (pH 6.0), 40 mM KCl, and 65 mM MnCl_2_ for a month. The resulting CENP-A-H3.3 nucleosome crystals were cryoprotected with a 30% polyethylene glycol 400 solution, containing 20 mM potassium cacodylate (pH 6.0), 40 mM KCl, 50 mM MnCl_2_, and 5% trehalose, and were flash-cooled in a stream of N_2_ gas (100 K). The diffraction data were collected at the beamline BL17A (wavelength: 0.97319 Å) at the Photon Factory (Tsukuba, Japan), and were processed using the HKL2000 and CCP4 programs^37^,^38^. The CENP-A/H3.3 nucleosome structure was determined by the molecular replacement method, with the human H3.1 nucleosome^39^ (PDB ID: 2CV5) as the search model, using the PHASER program^40^. Crystallographic refinement was performed using the PHENIX program^41^. The model rebuilding was performed using the Coot program^42^. All structural graphics were displayed using the PyMOL program (http://pymol.org). The atomic coordinates of the CENP-A/H3.3 nucleosome have been deposited in the Protein Data Bank, with the ID code 3WTP. In the refined model, 97.5% of the residues are in the favored regions of the Ramachandran plot, with 0.1% in the disallowed regions.

### MNase and exonuclease III treatment assays

The H3.3, CENP-A, or CENP-A/H3.3 nucleosomes (0.4 μM) were treated with MNase (Takara) or ExoIII (Takara). For the MNase assay, the nucleosome samples were incubated with MNase (0, 0.3, 0.5 and 0.7 units) for 5 minutes at 25°C in 10 μl of 44 mM Tris-HCl (pH 8.0) buffer, containing 15 mM NaCl, 2.5 mM CaCl_2_, and 1.9 mM dithiothreitol. After the MNase treatment, the reactions were stopped by the addition of 55 μl of 0.5 mg/ml proteinase K (Roche) solution, containing 20 mM Tris-HCl (pH 8.0), 20 mM EDTA, and 0.25% SDS. The resultant DNA fragments were extracted by phenol/chloroform/isoamyl alcohol, and were precipitated by ethanol. The purified DNA fragments were subjected to 10% native PAGE in 0.5 × TBE buffer (11.1 V/cm for 1 hr). For the ExoIII assay, the nucleosome samples were incubated with or without 2.0 units of ExoIII for 2.5, 5, and 7.5 minutes at 37°C in 10 μl of 63 mM Tris-HCl (pH 8.0) buffer, containing 5 mM MgCl_2_, 5 mM KCl, and 2.45 mM dithiothreitol. After the ExoIII treatment, the reactions were stopped by the addition of 55 μl of 0.5 mg/ml proteinase K (Roche) solution, containing 20 mM Tris-HCl (pH 8.0), 20 mM EDTA, and 0.25% SDS. The resultant DNA fragments were extracted by phenol/chloroform/isoamyl alcohol, and were precipitated by ethanol. The purified DNA fragments were subjected to 14% native PAGE in 0.5 × TBE buffer containing 7 M urea (11.1 V/cm for 3.5 hr).

### Thermal stability assay of nucleosomes

The stabilities of the H3.3, CENP-A, and CENP-A/H3.3 nucleosomes were evaluated by a thermal stability assay in the presence of SYPRO Orange, by the previously described method^29^,^30^. The thermal stability assay was performed in 10 μl of 20 mM Tris–HCl (pH 7.5) buffer, containing 1 mM dithiothreitol and 100 mM NaCl. The StepOnePlus™ Real-Time PCR unit (Applied Biosystems) was used to detect the fluorescence signals with a temperature gradient from 26°C to 95°C, in steps of 1°C/min. Raw fluorescence data were adjusted to normalized % values as (*F*(*T*) − *F*_26°C_)/(*F*_95°C_ − *F*_26°C_), where *F*(*T*), *F*_26°C_, and *F*_95°C_ indicate each fluorescence at a particular temperature, the fluorescence at 26°C, and the fluorescence at 95°C, respectively.

### Purification of the human CENP-C fragment

The DNA fragment encoding human CENP-C(426–537) was ligated between the *NdeI* and *BamHI* sites of the pET15b (Novagen) expression vector. The N-terminally His_6_-tagged CENP-C(426–537) fragment was expressed in *Escherichia coli* BL21(DE3) cells in the presence of isopropyl-β-D-thiogalactopyranoside (final concentration 1 mM). The cells producing the His_6_-tagged CENP-C(426–537) fragment were cultured overnight at 30°C. The cells were harvested and disrupted by sonication in 50 mM Tris–HCl (pH 8.0) buffer, containing 5% glycerol, 0.5 M NaCl, 1 mM phenylmethylsulfonyl fluoride, and 10 mM imidazole. His_6_-tagged CENP-C(426–537) was purified by Ni-NTA agarose chromatography (Qiagen), eluted with 50 mM Tris–HCl (pH 8.0) buffer, containing 5% glycerol, 0.5 M NaCl, 1 mM phenylmethylsulfonyl fluoride, and 500 mM imidazole. The His_6_-tag portion was removed by thrombin proteinase (GE Healthcare) during the dialysis step against 20 mM Tris-HCl (pH 7.5) buffer, containing 5% glycerol and 2 mM 2-mercaptoethanol, at 4°C overnight. CENP-C(426–537) was further purified by Mono S column chromatography (GE Healthcare), and was dialyzed against 50 mM Tris-HCl (pH 7.5) buffer, containing 100 mM NaCl, 5% glycerol, and 2 mM 2-mercaptoethanol. The purified CENP-C(426–537) was concentrated to 500 μM, and stored at −80°C.

### CENP-C binding assay

CENP-C(426–537) was mixed with the H3.3, CENP-A, or CENP-A/H3.3 nucleosomes (final concentration 0.2 μM), and incubated at 37°C for 30 minutes in 10 μl of 35 mM Tris-HCl (pH 7.5) buffer, containing 50 mM NaCl, 2.5% glycerol, 1 mM 2-mercaptoethanol, and 0.5 mM dithiothreitol. The final CENP-C(426–537) concentrations were 0, 0.2, 0.4, 0.6, 0.8, 1.0, and 1.2 μM. The samples were then subjected to 6% native PAGE in 0.2 × TBE buffer (8.3 V/cm for 1 hr).

## Author Contributions

Y.A. and K.S. reconstituted the CENP-A/H3.3 nucleosome, and performed structural and biochemical experiments. N.H. and Y.A. established the reconstitution method for the heterotypic nucleosome. R.F. purified the histones and CENP-A. H.T. established thermal stability assay, and K.S. and H.T. tested the thermal stability of the nucleosomes. Y.A., N.H. and W.K. collected X-ray diffraction data, and performed the structural analysis of the CENP-A/H3.3 nucleosome. T.F. and G.A. provided unpublished results for the CENP-A/H3.3 nucleosome formation in cells, and contributed to the initiation of this project. H.K. conceived, designed, and supervised all of the work, and H.K., G.A., T.F. and Y.A. wrote the paper. All of the authors discussed the results and commented on the manuscript.

## Additional Information

**Accession codes:** The atomic coordinates of the CENP-A/H3.3 nucleosome have been deposited in the Protein Data Bank, with the ID code 3WTP.

## Supplementary Material

Supplementary Information Supplementary figures
