# Supplementary material for: Crystal structure and stable property of the cancer-associated heterotypic nucleosome containing CENP-A and H3.3
Source: Sci Rep. 2014 Nov 19;4:7115. doi: 10.1038/srep07115 (PMC4236741; doi:10.1038/srep07115)
Supplement: Supplementary Information — Supplementary figures [file srep07115-s1.pdf]

Supplementary information

Crystal structure and stable property of the cancer-associated heterotypic nucleosome containing CENP-A and H3.3

Yasuhiro Arimura[1, 6], Kazuyoshi Shirayama[1, 6], Naoki Horikoshi[1], Risa Fujita[1], Hiroyuki Taguchi[1], Wataru Kagawa[1, 2], Tatsuo Fukagawa[3], Geneviève Almouzni[4, 5], and Hitoshi Kurumizaka[1,*]

[1] Laboratory of Structural Biology, Graduate School of Advanced Science and Engineering, Waseda University, 2-2 Wakamatsu-cho, Shinjuku-ku, Tokyo 162-8480, Japan.

[2] Program in Chemistry and Life Science, School of Science and Engineering, Meisei University, 2-1-1 Hodokubo, Hino-shi, Tokyo 191-8506, Japan.

[3] Department of Molecular Genetics, National Institute of Genetics, Mishima, Shizuoka 411-8540, Japan.

[4] Institut Curie, Centre de Recherche, Paris, F-75248 France.

[5] CNRS, UMR3664, Paris, F-75248 France.

[6] These authors equally contributed to this work.

[*]Address correspondence to:
Hitoshi Kurumizaka, Laboratory of Structural Biology, Graduate School of Advanced Science and Engineering, Waseda University, Shinjuku-ku, Tokyo 162-8480, Japan. Tel. +81-3-5369-7315; FAX. +81-3-5367-2820; E-mail: kurumizaka@waseda.jp

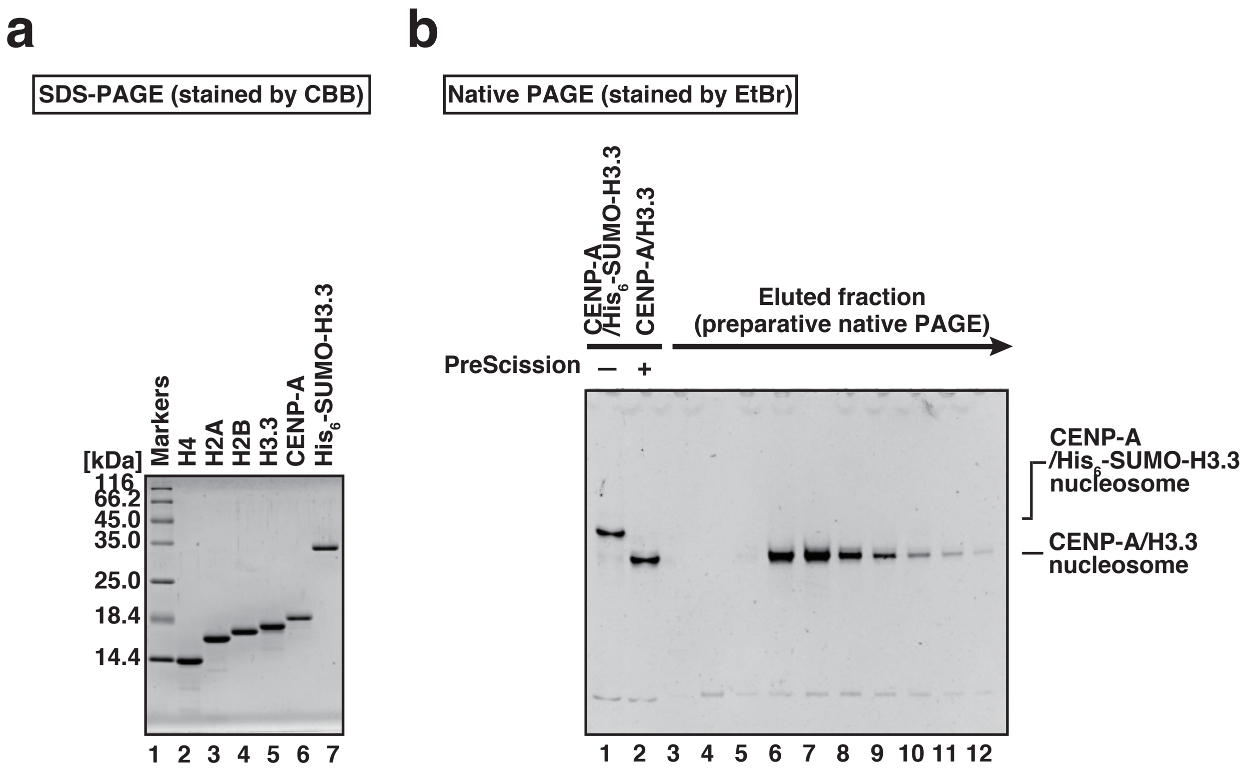

Supplementary Figure 1. Preparation of the CENP-A/His$_6$-SUMO-H3.3 nucleosome.

(**a**) Purified histones H4, H2A, H2B, H3.3, CENP-A, and His$_6$-SUMO-H3.3. Proteins were analyzed by 18% SDS-PAGE with Coomassie Brilliant Blue staining.

(**b**) The CENP-A/H3.3 nucleosome without the His$_6$-SUMO portion was purified by native PAGE with the Prep Cell apparatus.

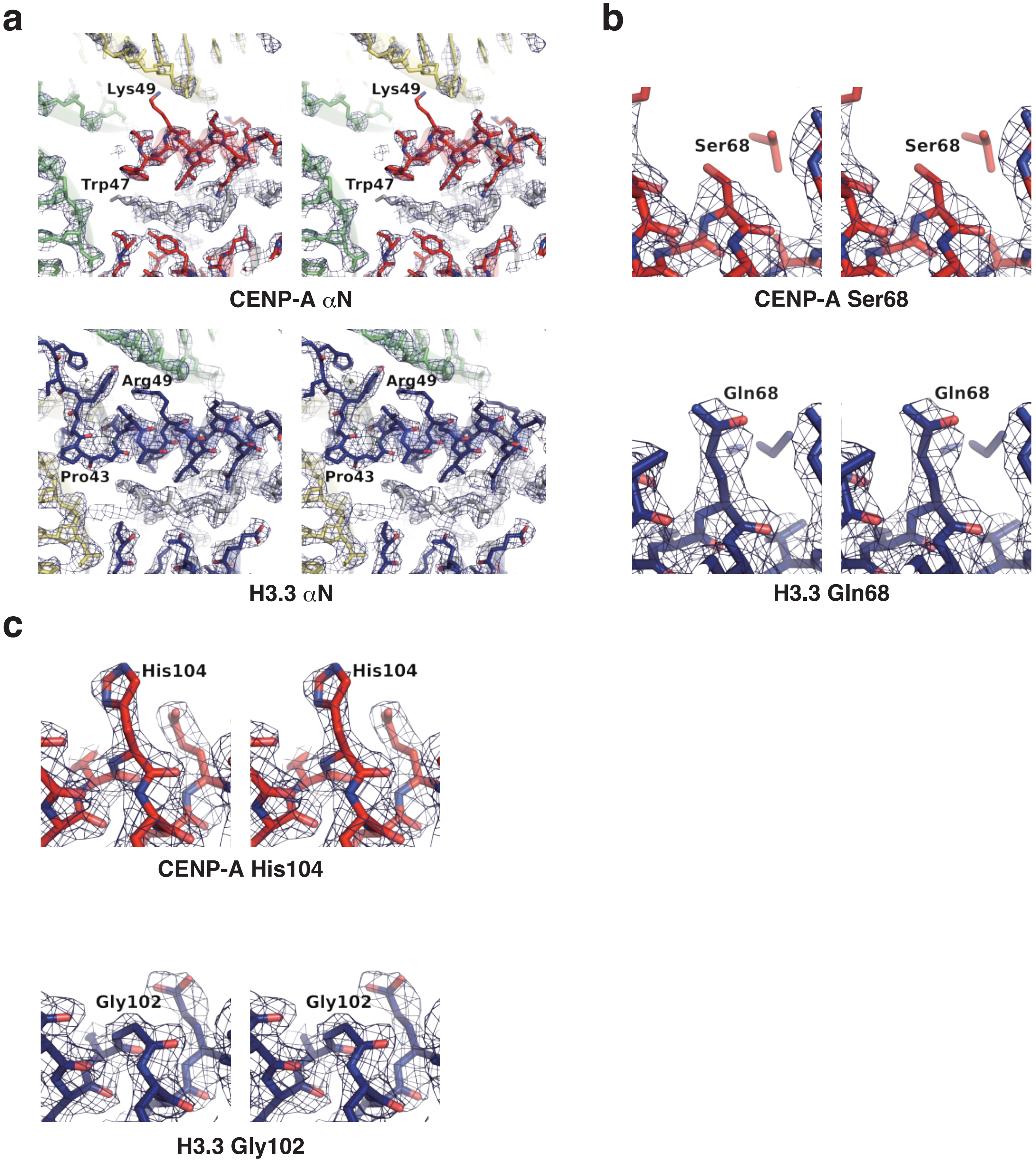

Supplementary Figure 2. Stereoviews of the electron density map of the CENP-A/H3.3 nucleosome. (**a**) The electron density map (blue mesh, contoured at 1.5σ) of the αN region of CENP-A (red, upper) and H3.3 (blue, lower). (**b**) The electron density map (blue mesh, contoured at 1.5σ) around the CENP-A Ser68 region (red, upper) and the H3.3 Gln68 region (blue, lower). (**c**) The electron density map (blue mesh, contoured at 1.5σ) of the CENP-A His104 region (red, upper) and the H3.3 Gly102 region (blue, lower).

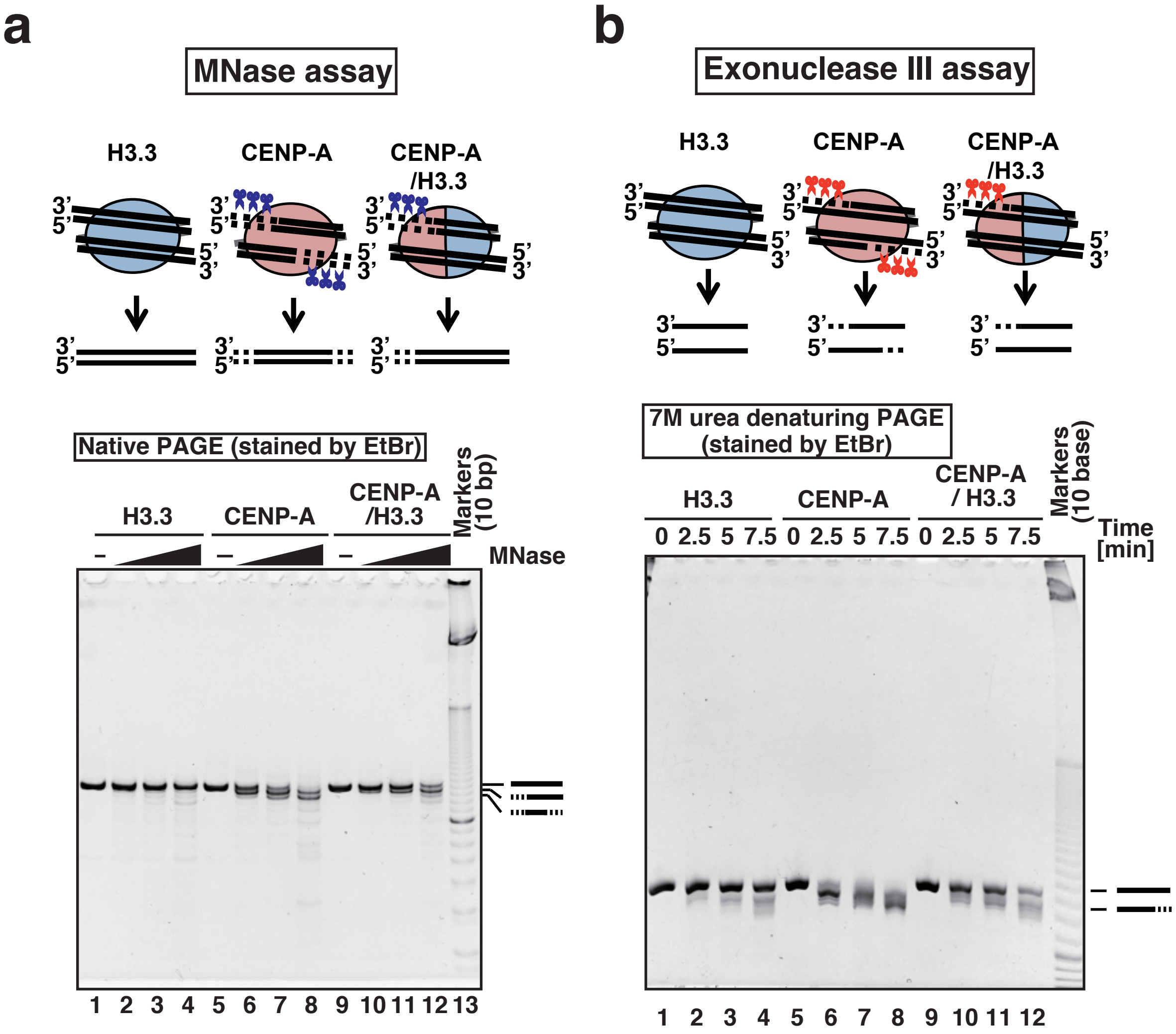

Supplementary Figure 3. MNase and ExoIII assays.

(**a**) Full image of the MNase assay, presented in Fig. 3a.

(**b**) Full image of the ExoIII assay, presented in Fig. 3b.

Supplementary Table 1 Data collection and refinement statistics

	CENP-A/H3.3 nucleosome
**Data collection**	
Space group	$P2_12_12_1$
Cell dimensions	
a, *b*, *c* (Å)	98.18, 107.67, 168.14
α, β, γ (°)	90.00, 90.00, 90.00
Resolution (Å)	50.0-2.67 (2.79-2.67)*
R_{sym} or R_{merge}	10.0 (38.1)
$I / \sigma I$	9.8(2.7)
Completeness (%)	98.0 (90.0)
Redundancy	5.5 (3.1)
**Refinement**	
Resolution (Å)	48.67-2.67
No. reflections	50779
R_{work} / R_{free}	22.85/26.99
No. atoms	
Protein	5936
DNA	5980
Water	-
B-factors	
Protein	34.62
DNA	76.10
Water	-
R.m.s. deviations	
Bond lengths (Å)	0.008
Bond angles (°)	1.005

*Values in parentheses are for highest-resolution shell.
